# Supplementary material for: Cellular senescence triggers intracellular acidification and lysosomal pH alkalinized via ATP6AP2 attenuation in breast cancer cells
Source: Commun Biol. 2023 Nov 22;6:1147. doi: 10.1038/s42003-023-05433-6 (PMC10665353; doi:10.1038/s42003-023-05433-6)
Supplement: Supplementary file 2 — Supplementary Information [file 42003_2023_5433_MOESM2_ESM.pdf]

Supplementary Information for

Cellular senescence triggers intracellular acidification and lysosomal pH alkalinized via ATP6AP2 attenuation in breast cancer cells

Wei Li[1], Kosuke Kawaguchi[1*], Sunao Tanaka[1], Chenfeng He[1], Yurina Maeshima[1], Eiji Suzuki[2], Masakazu Toi[1]

[1]Department of Breast Surgery, Kyoto University Graduate School of Medicine, 54 Shogoin-kawaharacho, Sakyo-ku, Kyoto 606-8507, Japan

[2]Kobe City Medical Center General Hospital, 2-1-1 Minatojimaminami-cho, Chuo-ku, Kobe 650-0047, Japan

Supplementary Fig. 1–3 is related to Fig. 1

Supplementary Fig. 4 is related to Fig. 2

Supplementary Fig. 5 is related to Fig. 3

Supplementary Fig. 6 is related to Fig. 4

Supplementary Fig. 7 is related to Fig. 5

Supplementary Fig. 8 is related to Fig. 6

Supplementary Fig. 9 is related to Fig. 7

Supplementary Fig. 10 is related to Supplementary Fig. 1 and 2

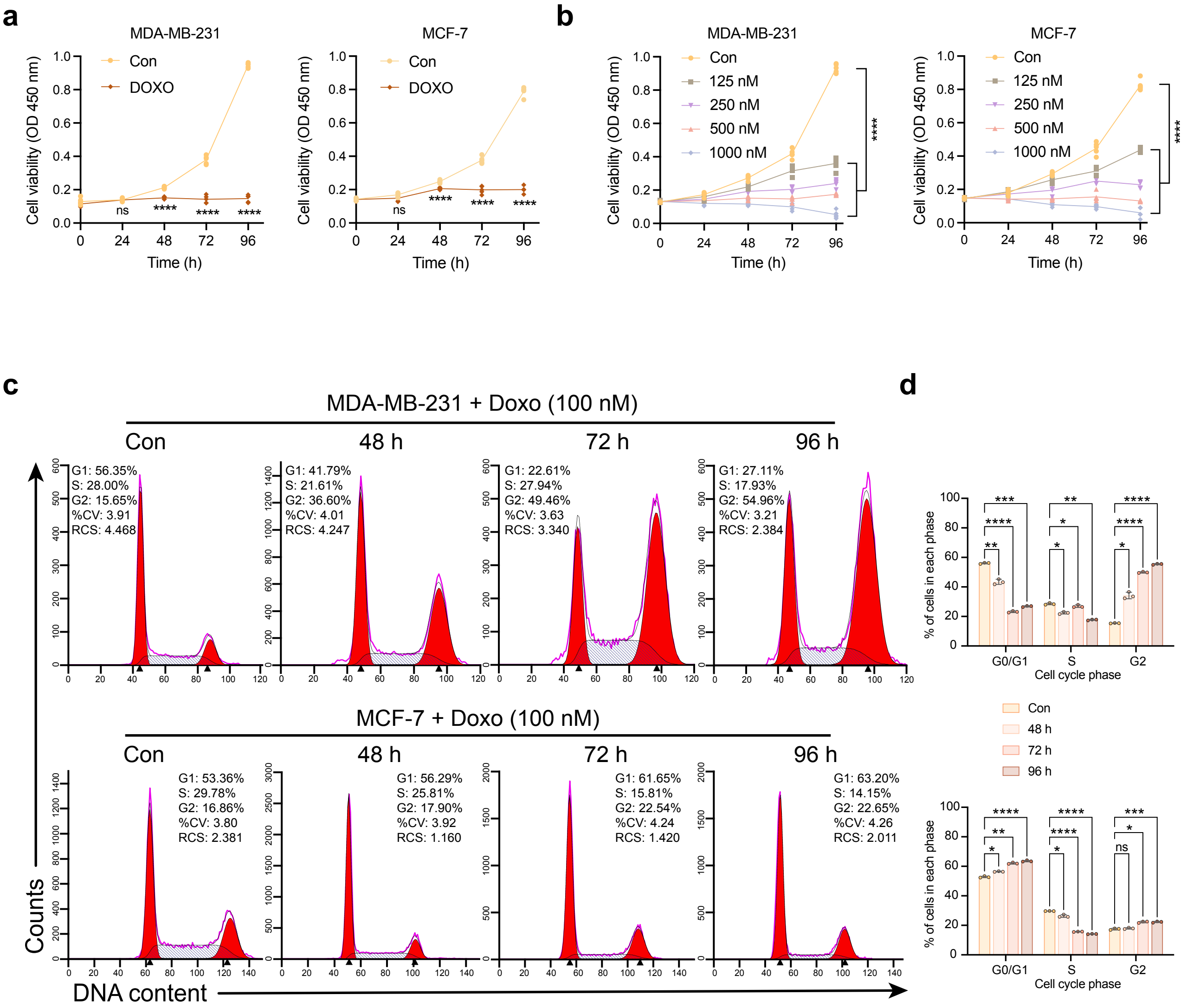

Supplementary Fig. 1. Doxo and Abe suppress breast cancer cell proliferation through cell cycle arrest.

a and **b** Cell proliferation in breast cancer cells treated with Doxo at 100 nM (**a**) and Abe at different concentrations (up to 1000 nM) (**b**) evaluated by the CCK-8 assay (n = 5).

c and **d** Representative images of the cell cycle analysis using propidium iodine (PI) detection by flow cytometry in Doxo (100 nM)-treated (**c**) breast cancer cells. Quantification of the percentage of cells in each cell cycle phase (**d**), respectively.

Data are presented as the means ± SD of three independent experiments. Statistical analyses were performed using two-way ANOVA with Sidak's multiple comparisons test (**a**), and Tukey's multiple comparisons test (**b** and **d**). ns, not significant; *$P < 0.05$; **$P < 0.01$; ***$P < 0.001$; ****$P < 0.0001$.

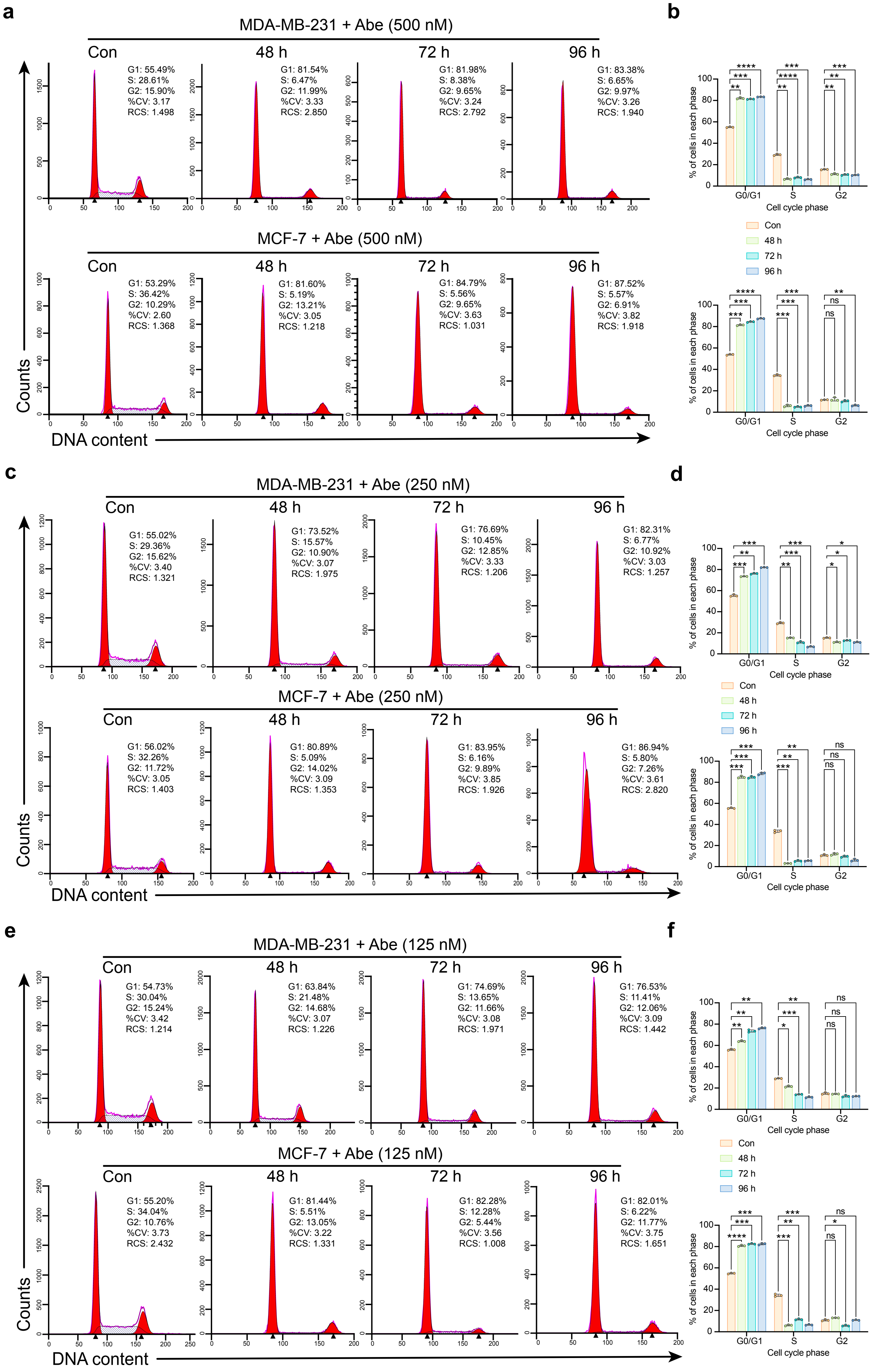

a
MDA-MB-231 + Abe (500 nM)
Con
G1: 55.49%
S: 28.61%
G2: 15.90%
%CV: 3.17
RCS: 1.498
48 h
G1: 81.54%
S: 6.47%
G2: 11.99%
%CV: 3.33
RCS: 2.850
72 h
G1: 81.98%
S: 8.38%
G2: 9.65%
%CV: 3.24
RCS: 2.792
96 h
G1: 83.38%
S: 6.65%
G2: 9.97%
%CV: 3.26
RCS: 1.940
MCF-7 + Abe (500 nM)
Con
G1: 53.29%
S: 36.42%
G2: 10.29%
%CV: 2.60
RCS: 1.368
48 h
G1: 81.60%
S: 5.19%
G2: 13.21%
%CV: 3.05
RCS: 1.218
72 h
G1: 84.79%
S: 5.56%
G2: 9.65%
%CV: 3.63
RCS: 1.031
96 h
G1: 87.52%
S: 5.57%
G2: 6.91%
%CV: 3.82
RCS: 1.918
Counts
DNA content
b
% of cells in each phase
Cell cycle phase
G0/G1
S
G2
Con
48 h
72 h
96 h
c
MDA-MB-231 + Abe (250 nM)
Con
G1: 55.02%
S: 29.36%
G2: 15.62%
%CV: 3.40
RCS: 1.321
48 h
G1: 73.52%
S: 15.57%
G2: 10.90%
%CV: 3.07
RCS: 1.975
72 h
G1: 76.69%
S: 10.45%
G2: 12.85%
%CV: 3.33
RCS: 1.206
96 h
G1: 82.31%
S: 6.77%
G2: 10.92%
%CV: 3.03
RCS: 1.257
MCF-7 + Abe (250 nM)
Con
G1: 56.02%
S: 32.26%
G2: 11.72%
%CV: 3.05
RCS: 1.403
48 h
G1: 80.89%
S: 5.09%
G2: 14.02%
%CV: 3.09
RCS: 1.353
72 h
G1: 83.95%
S: 6.16%
G2: 9.89%
%CV: 3.85
RCS: 1.926
96 h
G1: 86.94%
S: 5.80%
G2: 7.26%
%CV: 3.61
RCS: 2.820
Counts
DNA content
d
% of cells in each phase
Cell cycle phase
G0/G1
S
G2
Con
48 h
72 h
96 h
e
MDA-MB-231 + Abe (125 nM)
Con
G1: 54.73%
S: 30.04%
G2: 15.24%
%CV: 3.42
RCS: 1.214
48 h
G1: 63.84%
S: 21.48%
G2: 14.68%
%CV: 3.07
RCS: 1.226
72 h
G1: 74.69%
S: 13.65%
G2: 11.66%
%CV: 3.08
RCS: 1.971
96 h
G1: 76.53%
S: 11.41%
G2: 12.06%
%CV: 3.09
RCS: 1.442
MCF-7 + Abe (125 nM)
Con
G1: 55.20%
S: 34.04%
G2: 10.76%
%CV: 3.73
RCS: 2.432
48 h
G1: 81.44%
S: 5.51%
G2: 13.05%
%CV: 3.22
RCS: 1.331
72 h
G1: 82.28%
S: 12.28%
G2: 5.44%
%CV: 3.56
RCS: 1.008
96 h
G1: 82.01%
S: 6.22%
G2: 11.77%
%CV: 3.75
RCS: 1.651
Counts
DNA content
f
% of cells in each phase
Cell cycle phase
G0/G1
S
G2
Con
48 h
72 h
96 h

Supplementary Fig. 2. Abe causes cell cycle arrest at G0/G1 phase in breast cancer cells

a and **b** Representative images of the cell cycle analysis using PI detection by flow cytometry in Abe (500 nM)-treated (**a**) breast cancer cells. Quantification of the percentage of cells in each cell cycle phase (**b**), respectively.

c and **d** Representative images of the cell cycle analysis detected by flow cytometry in Abe (250 nM)-treated (**c**) breast cancer cells. Quantification of the percentage of cells in each cell cycle phase (**d**), respectively.

e and **f** Representative images of the cell cycle analysis detected by flow cytometry in Abe (125 nM)-treated (**e**) breast cancer cells. Quantification of the percentage of cells in each cell cycle phase (**f**), respectively.

Data are presented as the means ± SD of three independent experiments. Statistical analyses were performed by two-way ANOVA with Tukey’s multiple comparisons test (**b**), and Dunnett's multiple comparisons test (**d** and **f**). ns, not significant; $^{*}P < 0.05$; $^{**}P < 0.01$; $^{***}P < 0.001$; $^{****}P < 0.0001$.

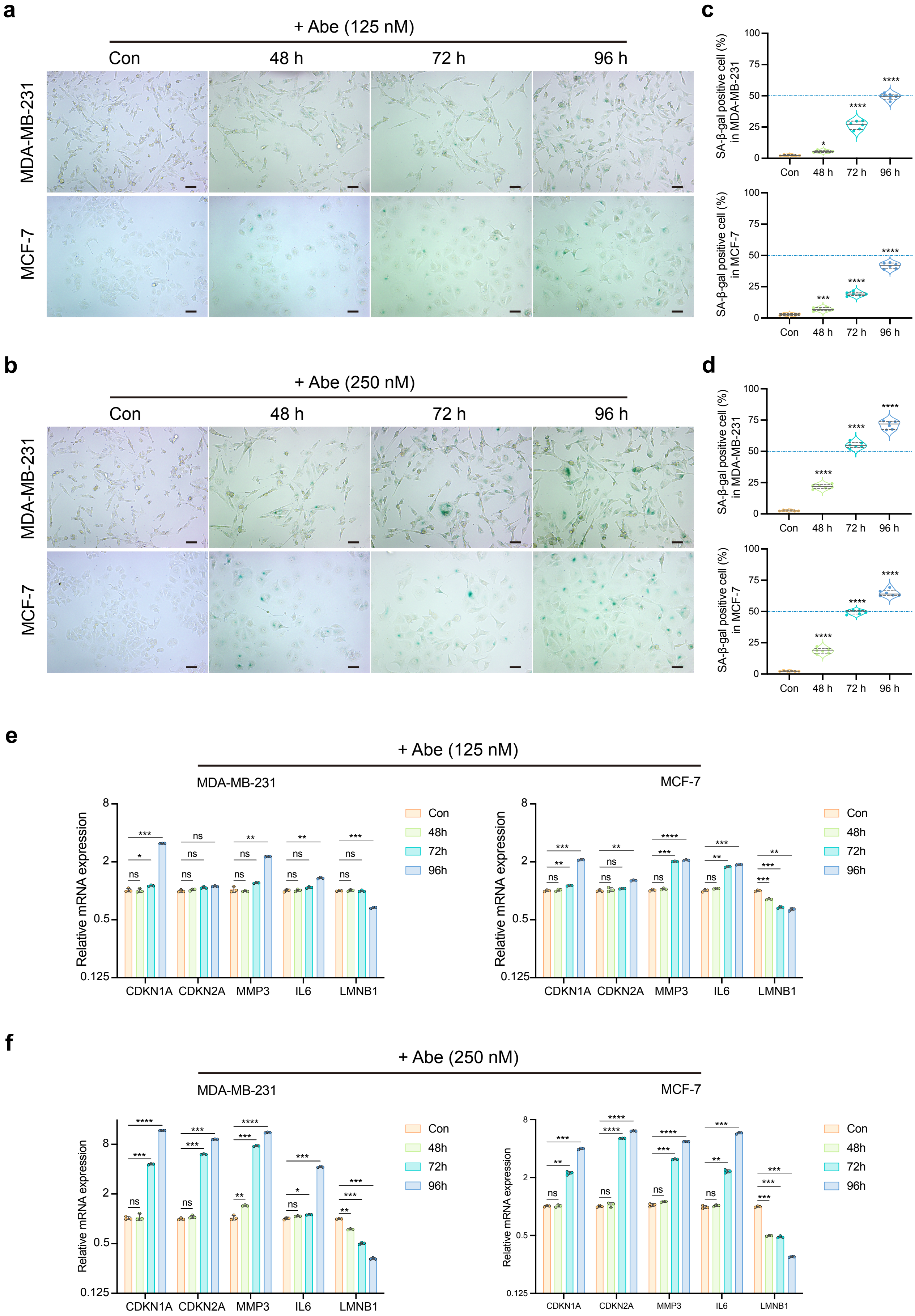

a
+ Abe (125 nM)
Con
48 h
72 h
96 h
MDA-MB-231
MCF-7
b
+ Abe (250 nM)
Con
48 h
72 h
96 h
MDA-MB-231
MCF-7
c
SA-β-gal positive cell (%) in MDA-MB-231
SA-β-gal positive cell (%) in MCF-7
d
SA-β-gal positive cell (%) in MDA-MB-231
SA-β-gal positive cell (%) in MCF-7
e
+ Abe (125 nM)
MDA-MB-231
MCF-7
Relative mRNA expression
CDKN1A
CDKN2A
MMP3
IL6
LMNB1
Con
48h
72h
96h
f
+ Abe (250 nM)
MDA-MB-231
MCF-7
Relative mRNA expression
CDKN1A
CDKN2A
MMP3
IL6
LMNB1
Con
48h
72h
96h

Supplementary Fig. 3. Abe elicits senescent phenotype in breast cancer cells.

a and **b** Representative SA-β-gal staining in Abe (125 nM)-treated (**a**) and Abe (250 nM)-treated (**b**) breast cancer cells.

c and **d** Quantitative analysis of the percentage of SA-β-gal positive cells in Abe (125 nM)-treated (**c**) and Abe (250 nM)-treated (**d**) breast cancer cells, respectively. At least six separate fields of view were taken.

e and **f** RT-qPCR analyzed mRNA relative expression levels of senescence-related genes (*CDKN1A, CDKN21, MMP3, IL6, LMNB1*) in Abe (125 nM)-treated (**e**) and Abe (250 nM)-treated (**f**) breast cancer cells, respectively.

Scale bars represent 50 μm. Data are shown as the means ± SD of three independent experiments. One-way ANOVA with Dunnett's multiple comparisons test (**c** and **d**) and two-way ANOVA with Dunnett's multiple comparisons test (**e** and **f**) were performed. ns, not significant; *$P < 0.05$; **$P < 0.01$; ***$P < 0.001$; ****$P < 0.0001$.

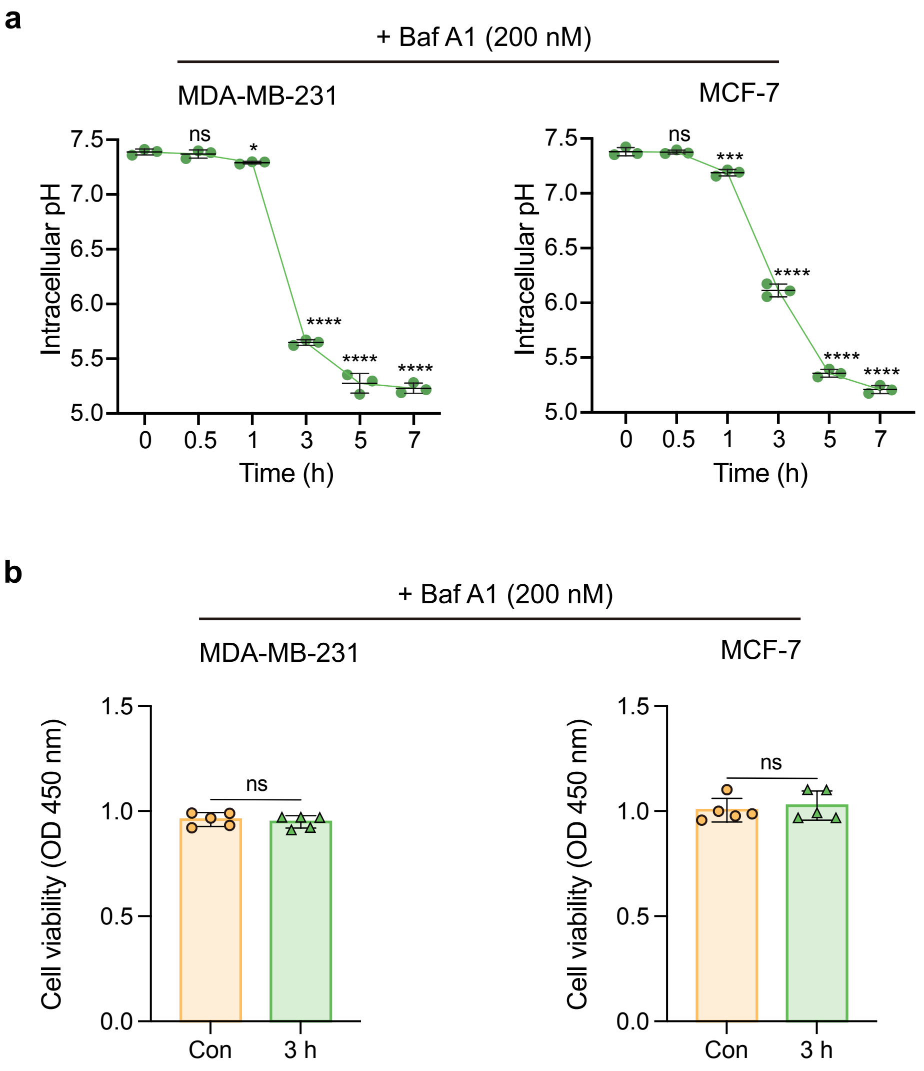

Supplementary Fig. 4. Baf A1 induces reduction in intracellular pH.

a Baf A1 (200 nM) regulates pH_i in a time-dependent manner.

b The CCK-8 assay determined cell viability in breast cancer cells treated with Baf A1 (200 nM) for 3 h.

Data are shown as the means ± SD of three independent experiments. One-way ANOVA with Dunnett's multiple comparisons test (**a**) and unpaired two-tailed Student's *t* test (**b**) were performed. ns, not significant; *$P < 0.05$; ***$P < 0.001$; ****$P < 0.0001$.

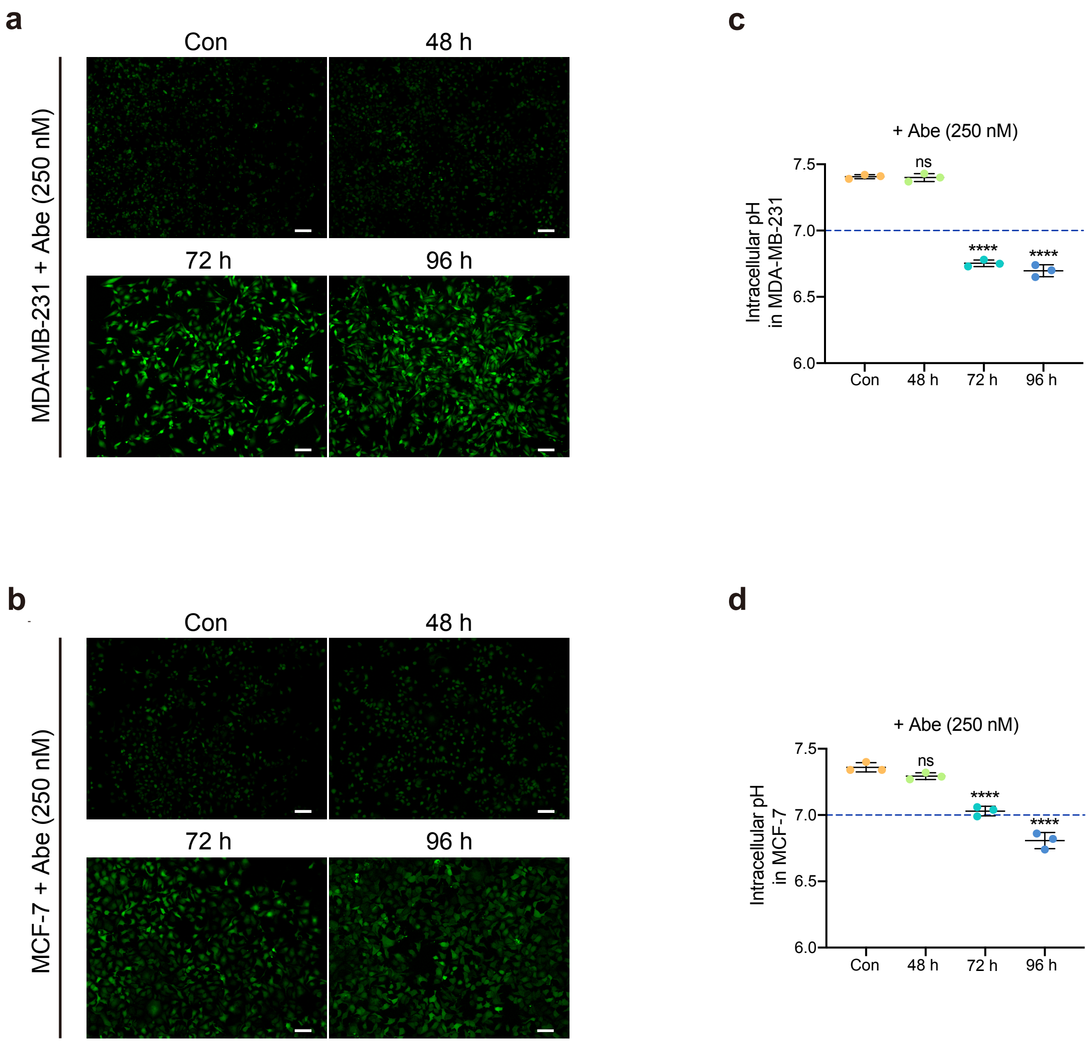

Supplementary Fig. 5. Abe induces intracellular pH (pH_i) decrease in breast cancer cells.

a and **b** Representative fluorescent images of Abe (250 nM)-treated MDA-MB-231 (**a**) and MCF-7 (**b**) stained with pHrodo Green AM.

c and **d** Quantitative analysis of the pH_i in MDA-MB-231 (**c**) and MCF-7 cells (**d**) treated with Abe (250 nM).

Scale bars represent 50 μm. Data are shown as the means ± SD of three independent experiments. One-way ANOVA with Dunnett's multiple comparisons test (**c** and **d**) was performed. ns, not significant; ****P < 0.0001.

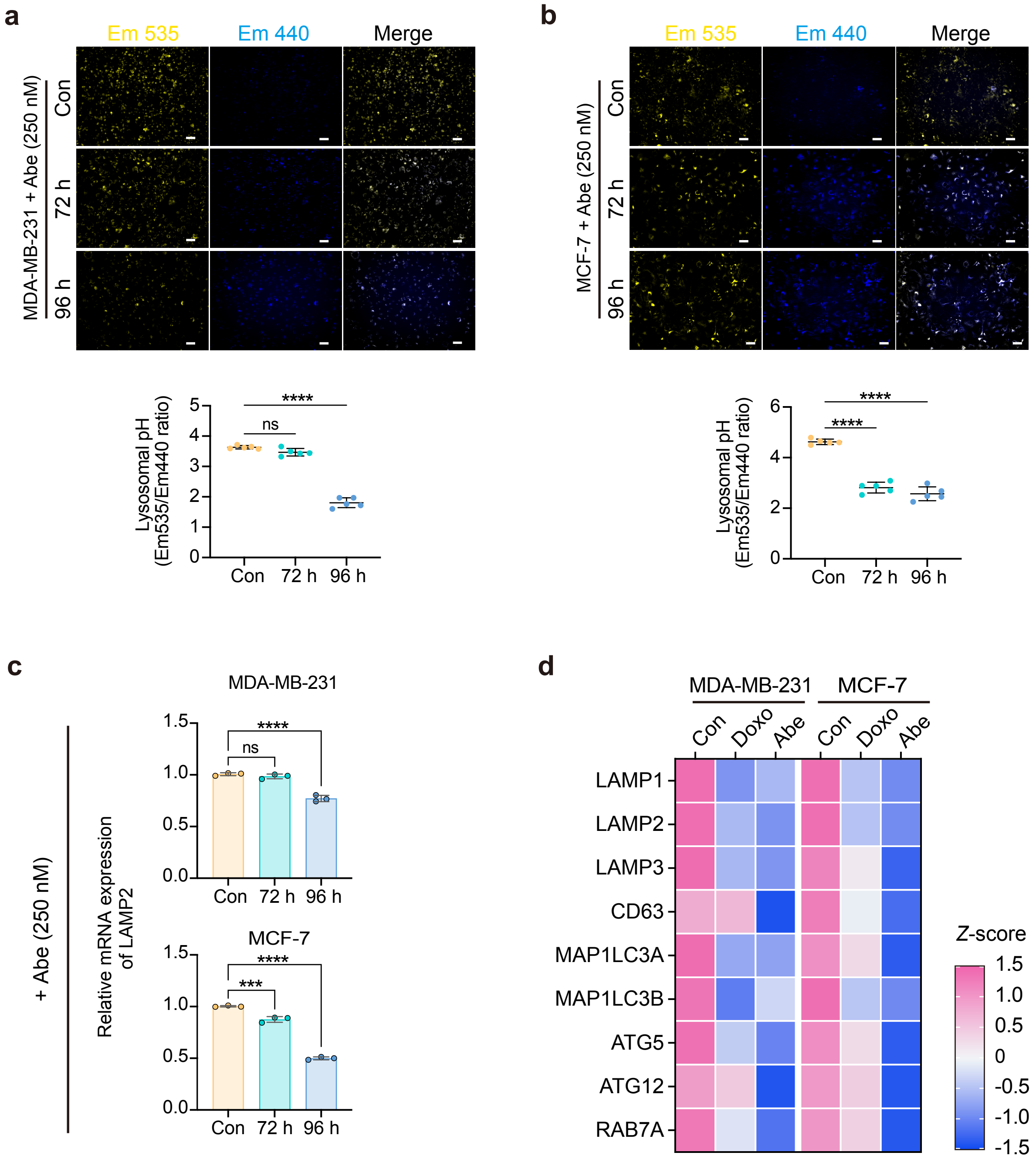

Supplementary Fig. 6. Doxo and Abe increase lysosomal pH (pH_L) and trigger lysosomal dysfunction.

a and **b** Representative fluorescent images of Abe (250 nM)-treated MDA-MB-231 (**a** upper) and MCF-7 (**b** upper) stained with LysoSensor Yellow/Blue-DND-160 to assess pH_L status. Em 535 (yellow), Em440 (blue). Quantitative analysis of the pH_L status (**a** and **b**, lower panel).

c RT-qPCR analyzed the mRNA relative expression levels of *LAMP2* in Abe (250nM)-treated breast cancer cells.

d Heatmap represents the standardized mRNA abundance values (*z*-score) of the lysosomal function-related genes.

Scale bars represent 50 μm. Data are shown as the means ± SD of three independent experiments. One-way ANOVA with Dunnett's multiple comparisons test (**a**, **b** lower and **c**) was performed. ns, not significant; ***P < 0.001; ****P < 0.0001.

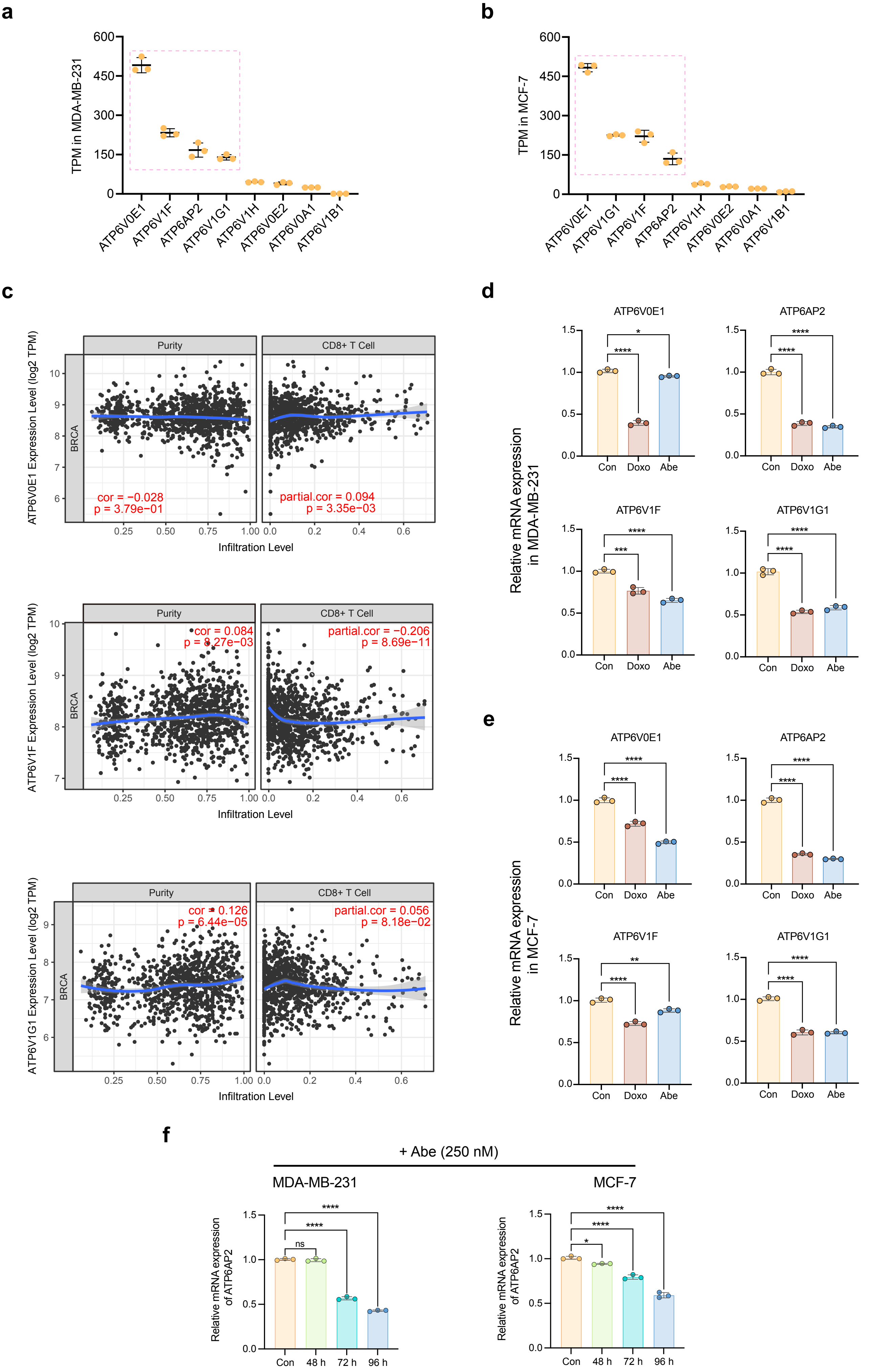

a
TPM in MDA-MB-231
600
450
300
150
0
ATP6V0E1
ATP6V1F
ATP6AP2
ATP6V1G1
ATP6V1H
ATP6V0E2
ATP6V0A1
ATP6V1B1
b
TPM in MCF-7
600
450
300
150
0
ATP6V0E1
ATP6V1G1
ATP6V1F
ATP6AP2
ATP6V1H
ATP6V0E2
ATP6V0A1
ATP6V1B1
c
Purity
CD8+ T Cell
ATP6V0E1 Expression Level (log2 TPM)
BRCA
cor = −0.028
p = 3.79e−01
partial.cor = 0.094
p = 3.35e−03
Infiltration Level
ATP6V1F Expression Level (log2 TPM)
cor = 0.084
p = 8.27e−03
partial.cor = −0.206
p = 8.69e−11
ATP6V1G1 Expression Level (log2 TPM)
cor = 0.126
p = 6.44e−05
partial.cor = 0.056
p = 8.18e−02
0.25
0.50
0.75
1.00
0.0
0.2
0.4
0.6
d
Relative mRNA expression in MDA-MB-231
ATP6V0E1
ATP6AP2
ATP6V1F
ATP6V1G1
Con
Doxo
Abe
e
Relative mRNA expression in MCF-7
f
+ Abe (250 nM)
MDA-MB-231
MCF-7
Relative mRNA expression of ATP6AP2
Con
48 h
72 h
96 h
ns
*
**
***
****

Supplementary Fig. 7. Validation of differentially expressed V-ATPase subunits and their correlation with CD8^{+} T cell infiltration.

a and **b** V-ATPase subunit expression in overlapping down-regulated genes in MDA-MB-231 (**a**) and MCF-7 (**b**). The pink dashed rectangle represents the top four V-ATPase subunits based on relative expression levels in RNA sequencing.

c Correlation of *ATP6V0E1*, *ATP6V1F*, and *ATP6V1G1* expression levels with CD8^{+} T cell infiltration level in The Cancer Genome Atlas Breast Invasive Carcinoma (TCGA-BRCA) dataset analyzed using theTumor Immune Estimation Resource (TIMER) database.

d and **e** RT-qPCR validated the relative expression levels of top four V-ATPase subunits (*ATP6V0E1, ATP6V1F, ATP6AP2, ATP6V1G1*) in MDA-MB-231 (**d**) and MCF-7 (**e**), respectively.

f RT-qPCR analyzed mRNA relative expression levels of *ATP6AP2* in breast cancer cells treated with Abe (250 nM).

Data are shown as the means ± SD of three independent experiments. One-way ANOVA with Dunnett's multiple comparisons test (**c**, **d**, and **e**) was performed. ns, not significant; *P < 0.05; **P < 0.01; ***P < 0.001; ****P < 0.0001.

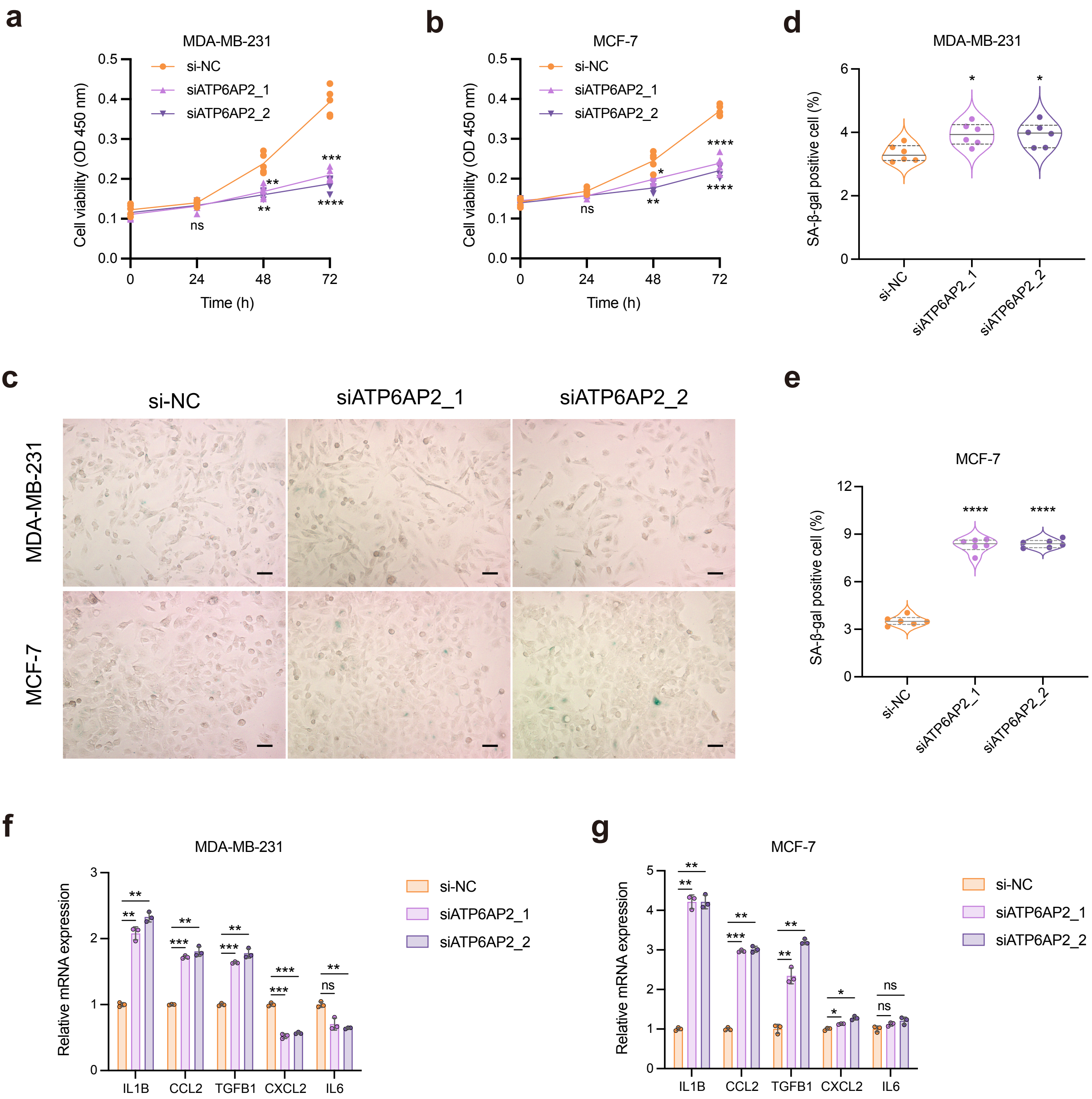

Supplementary Fig. 8. Downregulated ATP6AP2 facilitates cellular senescence phenotypes in breast cancer cells.

a and **b** *ATP6AP2* downregulation on cell proliferation was evaluated using CCK-8 analysis in MDA-MB-231 (**a**) and MCF-7 (**b**) cells, in comparison to the siRNA negative control (n = 5).

c Representative SA-β-gal staining in *ATP6AP2* downregulated breast cancer cells.

d and **e** Quantitative analysis of the percentage of SA-β-gal positive cells in MDA-MB-231 (**d**) and MCF-7 (**e**) cells, respectively. At least six separate fields of view were taken.

f and **g** RT-qPCR analyzed mRNA relative expression levels of genes of SASP (*IL1B, CCL2, TGFB1, CXCL2, IL6*) in MDA-MB-231 (**f**) and MCF-7 (**g**) cells, respectively.

Scale bars represent 50 μm. Data are shown as the means ± SD of three independent experiments. Two-way ANOVA with Dunnett's multiple comparisons test (**a, b, f** and **g**) and one-way ANOVA with Dunnett's multiple comparisons test (**d** and **e**) were performed. ns, not significant; *P < 0.05; **P < 0.01; ***P < 0.001; ****P < 0.0001.

a

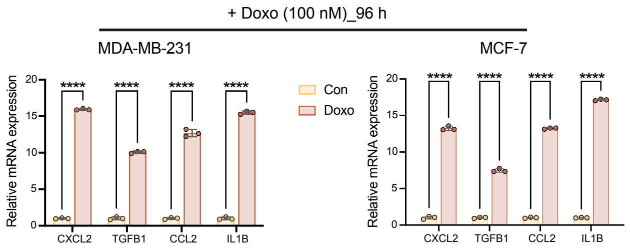

b

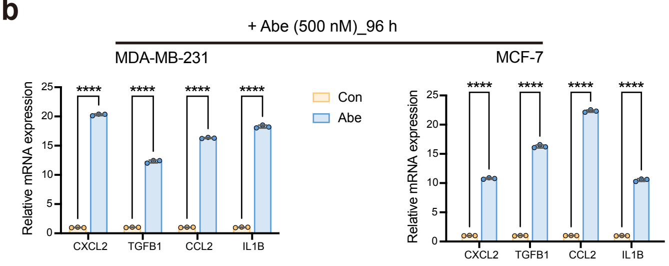

c

MDA-MB-231 + Doxo					
Gene set	EnrichmentScore	NES	pvalue	p.adjust	qvalue
HALLMARK_IL6_JAK_STAT3_SIGNALING	0.662286586	1.947916196	1.55E-07	3.88E-06	2.62E-06
HALLMARK_INFLAMMATORY_RESPONSE	0.573613859	1.780348534	5.72E-08	2.86E-06	1.93E-06
HALLMARK_TNFA_SIGNALING_VIA_NFKB	0.549519173	1.714536366	2.48E-07	4.13E-06	2.78E-06
HALLMARK_INTERFERON_GAMMA_RESPONSE	0.541852108	1.688129539	1.21E-06	1.30E-05	8.77E-06
HALLMARK_EPITHELIAL_MESENCHYMAL_TRANSITION	0.531013465	1.655589493	3.62E-06	3.02E-05	2.03E-05

MDA-MB-231 + Abe					
Gene set	EnrichmentScore	NES	pvalue	p.adjust	qvalue
HALLMARK_IL6_JAK_STAT3_SIGNALING	0.453436468	1.659477426	2.91E-03	1.12E-02	7.77E-03
HALLMARK_EPITHELIAL_MESENCHYMAL_TRANSITION	0.36331503	1.515126857	9.71E-04	4.05E-03	2.81E-03
HALLMARK_E2F_TARGETS	-0.619352523	-2.449780331	1.00E-10	2.50E-09	1.74E-09
HALLMARK_MYC_TARGETS_V1	-0.499106471	-1.969914649	6.51E-09	1.08E-07	7.53E-08
HALLMARK_DNA_REPAIR	-0.411991039	-1.572194417	8.86E-04	4.03E-03	2.80E-03

MCF-7 + Doxo					
Gene set	EnrichmentScore	NES	pvalue	p.adjust	qvalue
HALLMARK_EPITHELIAL_MESENCHYMAL_TRANSITION	0.694417423	2.234456966	1.00E-10	7.14E-10	3.01E-10
HALLMARK_INTERFERON_GAMMA_RESPONSE	0.69039349	2.221157618	1.00E-10	7.14E-10	3.01E-10
HALLMARK_INTERFERON_ALPHA_RESPONSE	0.720489535	2.163220483	1.00E-10	7.14E-10	3.01E-10
HALLMARK_TNFA_SIGNALING_VIA_NFKB	0.623348163	2.007212762	1.00E-10	7.14E-10	3.01E-10
HALLMARK_INFLAMMATORY_RESPONSE	0.611687987	1.940291504	2.16E-09	1.35E-08	5.69E-09

MCF-7 + Abe					
Gene set	EnrichmentScore	NES	pvalue	p.adjust	qvalue
HALLMARK_EPITHELIAL_MESENCHYMAL_TRANSITION	0.389706223	1.800624619	6.04E-06	7.55E-05	4.93E-05
HALLMARK_INFLAMMATORY_RESPONSE	0.368225664	1.689939715	3.90E-05	2.79E-04	1.82E-04
HALLMARK_TNFA_SIGNALING_VIA_NFKB	0.349760958	1.623568261	1.16E-04	6.45E-04	4.21E-04
HALLMARK_IL6_JAK_STAT3_SIGNALING	0.374818714	1.501281424	1.14E-02	3.55E-02	2.32E-02
HALLMARK_E2F_TARGETS	-0.705180112	-2.638908129	1.00E-10	2.50E-09	1.63E-09

Supplementary Fig. 9. Doxo and Abe promote expression of immunomodulatory factors and alter enriched pathways in breast cancer cells.

a and **b** RT-qPCR analyzed mRNA relative expression levels of immunomodulatory-related genes (*CXCL2, TGFB1, CCL2, IL1B*) in Doxo (100 nM)-treated (**a**) and Abe (500 nM)-treated (**b**) breast cancer cells, respectively.

c GSEA enrichment analysis was performed in Doxo (100 nM)-treated and Abe (500 nM)-treated breast cancer cells, respectively. The table shows the detailed results of the enriched pathways.

Data are shown as the means ± SD of three independent experiments. Two-way ANOVA with Sidak's multiple comparisons test (**a** and **b**) was performed. ****P < 0.0001.

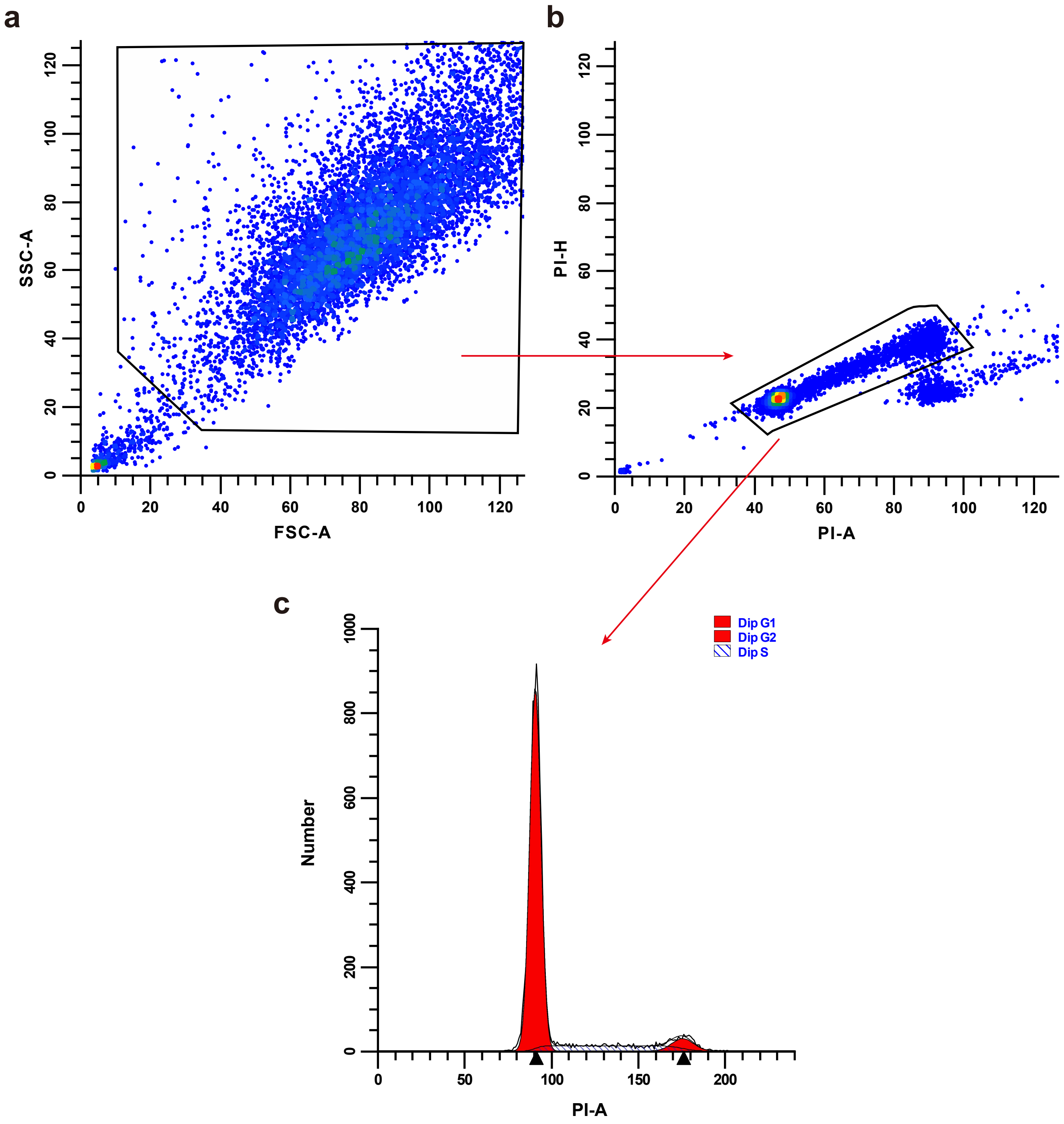

Supplementary Fig. 10. FACS gating strategy in propidium iodide staining cell cycle assay.

a Data were collected using BD FACSDiva Software v8.0.1. Cells were selected from cellular debris based on the FSC area/SSC area.

b Singlets then were selected based on the PI-FL2 area/ PI-FL2 Height.

c The cell cycle phase curve was fitted with ModFit software. And Reduced Chi-Square (RCS), a measure of goodness of fit, Values less than 5.0.

Supplementary Table 1

Group	MDA-MB-231 (TPM)			MCF-7 (TPM)		
	231-Con	231-Doxo	231-Abe	7-Con	7-Doxo	7-Abe
**LAMP1**	254.8058	75.95875	100.4852	132.6199	61.99424	42.95981
**LAMP2**	164.0145	79.34554	64.82179	51.30565	24.03746	17.01098
**LAMP3**	0.512301	0.157638	0.104327	3.160298	2.218661	0.979365
**CD63**	433.934	424.0611	256.0624	459.6124	371.0348	294.0614
**MAP1LC3A**	13.57517	6.84313	6.679199	0.590627	0.438305	0.118992
**MAP1LC3B**	6.044111	3.629767	4.43065	75.12739	62.5088	58.89508
**ATG5**	28.19634	23.09392	21.26248	28.06269	24.01569	16.14677
**ATG12**	30.48079	30.41372	30.14027	29.47602	28.84029	26.70154
**RAB7A**	257.5688	209.4985	176.5374	256.4669	249.5	229.1101

Group	MDA-MB-231				
	Average	SD	z-score		
**LAMP1**	143.7499	79.16414	1.402856	-0.856337	-0.546519
**LAMP2**	102.7273	43.74033	1.40116	-0.534557	-0.866602
**LAMP3**	0.258089	0.181068	1.403961	-0.55477	-0.849191
**CD63**	371.3525	81.62199	0.766724	0.645765	-1.412488
**MAP1LC3A**	9.032501	3.212853	1.413907	-0.681442	-0.732465
**MAP1LC3B**	4.701509	1.004088	1.337136	-1.067379	-0.269757
**ATG5**	24.18425	2.933852	1.367518	-0.371638	-0.995881
**ATG12**	30.34493	0.147285	0.922474	0.467085	-1.389559
**RAB7A**	214.5349	33.27204	1.293394	-0.15137	-1.142024

Group	MDA-MB-231				
	Average	SD	z-score		
**LAMP1**	79.19133	38.57062	1.385215	-0.44586	-0.939355
**LAMP2**	30.7847	14.79133	1.387364	-0.456162	-0.931202
**LAMP3**	2.119441	0.893122	1.165413	0.111094	-1.276507
**CD63**	374.9029	67.64126	1.252336	-0.057185	-1.195151
**MAP1LC3A**	0.382642	0.196526	1.058312	0.283236	-1.341549
**MAP1LC3B**	65.51043	6.958414	1.382063	-0.431366	-0.950697
**ATG5**	22.74172	4.94736	1.075518	0.257505	-1.333023
**ATG12**	28.33928	1.186785	0.957828	0.422155	-1.379982
**RAB7A**	245.0257	11.60785	0.985646	0.385458	-1.371104

Supplementary Table 1. RNA-seq normalization results for lysosome-related genes in 29 overlapped downregulated genes.

Supplementary Table 2

Group	MDA-MB-231 (TPM)			MCF-7 (TPM)		
	231-Con	231-Doxo	231-Abe	7-Con	7-Doxo	7-Abe
**ATP6V0E1**	491.2237	230.3726	234.0847	483.068	223.2373	157.2103
**ATP6V1F**	233.3758	109.5692	99.95342	221.4649	76.48275	85.15201
**ATP6AP2**	167.141	62.8521	65.97617	135.0201	54.24743	54.52637
**ATP6V1G1**	139.5136	53.32301	64.6393	225.3341	110.5389	103.3351
**ATP6V1H**	45.47745	17.56449	20.28437	39.5637	13.18008	14.08679
**ATP6V0E2**	40.33683	19.86086	15.44662	28.86607	6.111483	8.635479
**ATP6V0A1**	24.6106	11.18999	11.11805	21.62104	8.411995	6.514685
**ATP6V1B1**	0.837144	0.27683	0.354183	10.20945	3.942186	4.789519

Group	MDA-MB-231				
	Average	SD	z-score		
**ATP6V0E1**	318.5603	122.1009	1.414105	-0.722253	-0.691851
**ATP6V1F**	147.6328	60.75638	1.411258	-0.626495	-0.784763
**ATP6AP2**	98.65643	48.44271	1.413723	-0.739107	-0.674617
**ATP6V1G1**	85.82532	38.24344	1.403857	-0.849879	-0.553978
**ATP6V1H**	27.77544	12.56637	1.408682	-0.812562	-0.59612
**ATP6V0E2**	25.21477	10.84371	1.394547	-0.493734	-0.900813
**ATP6V0A1**	15.63955	6.343562	1.414198	-0.701429	-0.71277
**ATP6V1B1**	0.489386	0.247922	1.402694	-0.85735	-0.545344

Group	MCF-7				
	Average	SD	z-score		
**ATP6V0E1**	287.8385	140.6551	1.388001	-0.459288	-0.928713
**ATP6V1F**	127.6999	66.39627	1.412203	-0.771386	-0.640817
**ATP6AP2**	81.26465	38.01105	1.414207	-0.710773	-0.703434
**ATP6V1G1**	146.4027	55.89035	1.412254	-0.641681	-0.770573
**ATP6V1H**	22.27686	12.22925	1.413566	-0.743854	-0.669711
**ATP6V0E2**	14.53768	10.18397	1.406956	-0.827398	-0.579558
**ATP6V0A1**	12.18257	6.7188	1.404784	-0.561198	-0.843586
**ATP6V1B1**	6.313719	2.776333	1.403193	-0.854196	-0.548997

Supplementary Table 2. RNA-seq normalization results for representative subunits of V-ATPase in 29 overlapped downregulated genes.

Supplementary Table 3

Group	MDA-MB-231 (TPM)			MCF-7 (TPM)		
	231-Con	231-Doxo	231-Abe	7-Con	7-Doxo	7-Abe
**IL1A**	1.041938	13.23502	13.70842	0.047229	0.109261	0.104689
**IL1B**	1.75634	10.90239	15.37293	0.044085	0.170377	0.116485
**IL6**	20.3943	168.9246	69.61748	0.039469	0.768743	0.250759
**CCL2**	1.288564	3.270175	4.423768	0.103827	0.316935	0.437487
**CCL5**	1.326898	4.118867	4.263787	1.189923	7.289544	10.12159
**CXCL2**	6.428843	81.91512	121.4013	0.081051	0.392165	0.342842
**CXCL8**	60.60142	147.2887	187.0628	0.057282	0.587602	0.346597
**CXCL10**	0.439703	7.147013	5.834653	0.038827	1.606925	0.135234
**TGFB1**	87.40451	206.7277	204.0008	10.1757	24.62314	28.43897
**TGFB2**	25.75662	53.76866	59.4323	5.034845	10.14993	16.38193
**TNFSF10**	55.95949	125.6745	137.2826	7.054577	25.9531	42.81299
**TNFSF15**	6.08423	41.41327	39.10266	0.096319	0.803825	0.1533
**MMP3**	0.086773	3.095388	2.991923	0.03592	0.3155	0.161222
**CGAS**	26.09697	60.89907	123.4569	8.696542	25.9113	24.04705
**STING1**	36.83351	87.00439	95.9179	1.630873	4.635319	5.433757
**STAT3**	49.17544	118.2252	123.8268	47.42352	111.4084	111.984
**NFKB1**	19.29642	49.29644	41.49849	31.1816	71.24776	74.47357
**NFKB2**	43.92448	108.6238	96.17982	10.42714	23.78766	29.42708

Group	MDA-MB-231				
	Average	SD	z-score		
**IL1A**	9.328459	5.862641	-1.413445	0.666349	0.747096
**IL1B**	9.343889	5.667134	-1.338869	0.275007	1.063861
**IL6**	86.31214	61.77567	-1.067052	1.337298	-0.270247
**CCL2**	2.994169	1.294736	-1.317338	0.213175	1.104163
**CCL5**	3.236517	1.3516	-1.412858	0.652818	0.76004
**CXCL2**	69.91507	47.69811	-1.331001	0.251583	1.079418
**CXCL8**	131.651	52.79852	-1.345674	0.296178	1.049496
**CXCL10**	4.47379	2.902409	-1.38991	0.921036	0.468874
**TGFB1**	166.0443	55.6179	-1.41393	0.73148	0.68245
**TGFB2**	46.31919	14.72263	-1.396664	0.505987	0.890677
**TNFSF10**	106.3055	35.91405	-1.401848	0.539314	0.862533
**TNFSF15**	28.86672	16.13725	-1.411795	0.77749	0.634305
**MMP3**	2.058028	1.394528	-1.413565	0.743879	0.669686
**CGAS**	70.151	40.28184	-1.093645	-0.22968	1.323325
**STING1**	73.25193	26.00755	-1.400302	0.528787	0.871515
**STAT3**	97.07582	33.94779	-1.411001	0.622998	0.788004

**NFKB1**	36.69712	12.70932	-1.369129	0.991345	0.377783
**NFKB2**	82.90938	28.0307	-1.390793	0.917368	0.473425

**Group**	**MCF-7**				
	Average	SD	z-score		
**IL1A**	0.08706	0.028226	-1.411119	0.786543	0.624576
**IL1B**	0.110316	0.051742	-1.279997	1.160772	0.119225
**IL6**	0.35299	0.306375	-1.023324	1.357006	-0.333682
**CCL2**	0.286083	0.137952	-1.321155	0.223643	1.097512
**CCL5**	6.200352	3.726788	-1.344436	0.29226	1.052176
**CXCL2**	0.272019	0.136528	-1.398748	0.880007	0.518741
**CXCL8**	0.330494	0.216801	-1.260192	1.185917	0.074276
**CXCL10**	0.593662	0.717565	-0.773219	1.412085	-0.638865
**TGFB1**	21.07927	7.865789	-1.386201	0.450542	0.935659
**TGFB2**	10.52224	4.639904	-1.182652	-0.08024	1.262892
**TNFSF10**	25.27356	14.60622	-1.247344	0.046524	1.20082
**TNFSF15**	0.351148	0.320935	-0.79402	1.410494	-0.616473
**MMP3**	0.170881	0.114342	-1.180322	1.264794	-0.084473
**CGAS**	19.55163	7.713348	-1.407312	0.824502	0.58281
**STING1**	3.899983	1.637278	-1.385904	0.449121	0.936783
**STAT3**	90.27195	30.29933	-1.414171	0.697587	0.716584
**NFKB1**	58.96764	19.69178	-1.411047	0.623616	0.787431
**NFKB2**	21.21396	7.967322	-1.353882	0.323031	1.030851

Supplementary Table 3. RNA-seq normalization results for immunomodulatory-related genes of V-ATPase in 65 overlapped upregulated genes.

Supplementary Table 4

Gene	Forward	Reverse
CDKN1A	GGATGTCCGTCAGAACCC	GCTCCCAGGCGAAGTCA
CDKN2A	ATGGAGCCTTCGGCTGACT	GTAACTATTCGGTGCGTTGGG
IL6	TAGTCCTTCCTACCCCAATTTCC	TTGGTCCTTAGCCACTCCTTC
IL1B	CCACAGACCTTCCAGGAGAATG	GTGCAGTTCAGTGATCGTACAGG
MMP3	CTGGACTCCGACACTCTGGA	CAGGAAAGGTTCTGAAGTGACC
LMNB1	GAAAAAGACAACTCTCGTCGCA	GTAAGCACTGATTTCCATGTCCA
ATP6AP2	AGGCAGTGTCATTTCGTACC	GCCTTCCCTACCATATACACTC
ATP6V0E1	GTCCTAACCGGGGAGTTATCA	AAAGAGAGGGTTGAGTTGGGC
ATP6V1F	CTCATCGCAGTGATCGGAGAC	CGGTTCTTGTTAAGCTCCCCTAT
ATP6V1G1	CTAGTCAGTCTCAGGGGATTCA	GTTCTGCCGGAAGTATGTCTG
LAMP2	GAAAATGCCACTTGCCTTTATGC	AGGAAAAGCCAGGTCCGAAC
CXCL2	GGGCAGAAAGCTTGTCTCAA	GCTTCCTCCTTCCTTCTGGT
CCL2	AGAATCACCAGCAGCAAGTGTCC	TCCTGAACCCACTTCTGCTTGG
TGFB1	TACCTGAACCCGTGTTGCTCTC	GTTGCTGAGGTATCGCCAGGAA
ACTB	ATTGGCAATGAGCGGTTC	GGATGCCACAGGACTCCAT

Supplementary Table 4. Sequences of qRT-PCR primers. Sequences are represented as 5’ to 3’.
