# Supplementary material for: Cellular senescence triggers intracellular acidification and lysosomal pH alkalinized via ATP6AP2 attenuation in breast cancer cells
Source: Commun Biol. 2023 Nov 22;6:1147. doi: 10.1038/s42003-023-05433-6 (PMC10665353; doi:10.1038/s42003-023-05433-6)
Supplement: Supplementary file 3 — Description of Additional Supplementary Files [file 42003_2023_5433_MOESM3_ESM.pdf]

Description of Additional Supplementary Files

File name: Supplementary Data 1
Description: Differential expression of genes between Doxo (100 nM) -treated MDA-MB-231 cells for 96 h vs. control.

File name: Supplementary Data 2
Description: Differential expression of genes between Abe (500 nM) -treated MDA-MB-231 cells for 96 h vs. control.

File name: Supplementary Data 3
Description: Differential expression of genes between Doxo (100 nM) -treated MCF-7 cells for 96 h vs. control.

File name: Supplementary Data 4
Description: Differential expression of genes between Abe (500 nM) -treated MCF-7 cells for 96 h vs. control.

File name: Supplementary Data 5
Description: Source data underlying the graphs and charts presented in the main figures.
