# Supplementary material for: Cellular senescence triggers intracellular acidification and lysosomal pH alkalinized via ATP6AP2 attenuation in breast cancer cells
Source: Commun Biol. 2023 Nov 22;6:1147. doi: 10.1038/s42003-023-05433-6 (PMC10665353; doi:10.1038/s42003-023-05433-6)
Supplement: Supplementary file 9 — Reporting Summary [file 42003_2023_5433_MOESM9_ESM.pdf]

Corresponding author(s): Kosuke Kawaguchi, M.D., Ph.D.

Last updated by author(s): Oct 3, 2023

Reporting Summary

Nature Portfolio wishes to improve the reproducibility of the work that we publish. This form provides structure for consistency and transparency in reporting. For further information on Nature Portfolio policies, see our Editorial Policies and the Editorial Policy Checklist.

Statistics

For all statistical analyses, confirm that the following items are present in the figure legend, table legend, main text, or Methods section.

n/a	Confirmed	
☐	☒	The exact sample size (*n*) for each experimental group/condition, given as a discrete number and unit of measurement
☐	☒	A statement on whether measurements were taken from distinct samples or whether the same sample was measured repeatedly
☐	☒	The statistical test(s) used AND whether they are one- or two-sided<br>*Only common tests should be described solely by name; describe more complex techniques in the Methods section.*
☒	☐	A description of all covariates tested
☐	☒	A description of any assumptions or corrections, such as tests of normality and adjustment for multiple comparisons
☐	☒	A full description of the statistical parameters including central tendency (e.g. means) or other basic estimates (e.g. regression coefficient) AND variation (e.g. standard deviation) or associated estimates of uncertainty (e.g. confidence intervals)
☐	☒	For null hypothesis testing, the test statistic (e.g. *F*, *t*, *r*) with confidence intervals, effect sizes, degrees of freedom and *P* value noted<br>*Give P values as exact values whenever suitable.*
☒	☐	For Bayesian analysis, information on the choice of priors and Markov chain Monte Carlo settings
☒	☐	For hierarchical and complex designs, identification of the appropriate level for tests and full reporting of outcomes
☐	☒	Estimates of effect sizes (e.g. Cohen's *d*, Pearson's *r*), indicating how they were calculated

Our web collection on statistics for biologists contains articles on many of the points above.

Software and code

Policy information about availability of computer code

Data collection	Cell cycle distribution was detected by BD LSRFortessa Cell Analyzer<br>Absorbance value was detected by SpectraMax 340PC 384 Microplate Reader<br>Fluorescence intensity was detected by SpectraMax GEMINI EM<br>Images were acquired using KEYENCE BZ-X800 fluorescence microscope<br>RT-qPCR assays were performed by StepOnePlus Real-Time PCR System<br>RNA sequencing was sequenced with the TruSeq stranded mRNA Library on a NovaSeq6000 platform (Illumina)
Data analysis	Data analyzes were performed using the following software:<br>GraphPad Prism software v9.4.1<br>BD FACSDiva Software v8.0.1<br>ModFit LT software v5.0<br>Image J software v1.52<br>FastQC v0.11.9<br>Galaxy v22.05.1 https://usegalaxy.org<br>HISAT2 v2.2.1<br>Bioconductor v3.12.0<br>DAVID https://david.ncifcrf.gov<br>GSEA software v4.3.2<br>MSigDB v2022.1<br>STRING v11.5 https://string-db.org/

Cytoscape software v3.9.1
DESeq2 R package (v1.40.2)
ggplot2 R package (v3.4.2)
FactoMineR R package (v2.8)
VennDiagram R package (v1.7.3)
clusterProfiler R package(v4.8.2)
ComplexHeatmap R package (v2.16.0)
ggsankey R package (https://github.com/davidsjoberg/ggsankey)
RStudio software (v2023.06.0+421, RStudio, MA, USA) on the R computing environment (v4.3.1)
Excel for Mac Microsoft v16.69.1

For manuscripts utilizing custom algorithms or software that are central to the research but not yet described in published literature, software must be made available to editors and reviewers. We strongly encourage code deposition in a community repository (e.g. GitHub). See the Nature Portfolio guidelines for submitting code & software for further information.

Data

Policy information about availability of data

All manuscripts must include a data availability statement. This statement should provide the following information, where applicable:

- Accession codes, unique identifiers, or web links for publicly available datasets
- A description of any restrictions on data availability
- For clinical datasets or third party data, please ensure that the statement adheres to our policy

The RNA-Seq data generated in this study were deposited in the Gene Expression Omnibus (GEO) database under the accession code GSE222984. The remaining data are available in the article and supplementary data. Any other data supporting the findings of this study can be made available upon request from the corresponding authors. Source data for generating graphs and charts in the main figures are reported in Supplementary Data 5.

Research involving human participants, their data, or biological material

Policy information about studies with human participants or human data. See also policy information about sex, gender (identity/presentation), and sexual orientation and race, ethnicity and racism.

Reporting on sex and gender	Non
Reporting on race, ethnicity, or other socially relevant groupings	Non
Population characteristics	Non
Recruitment	Non
Ethics oversight	Non

Note that full information on the approval of the study protocol must also be provided in the manuscript.

Field-specific reporting

Please select the one below that is the best fit for your research. If you are not sure, read the appropriate sections before making your selection.

☒ Life sciences ☐ Behavioural & social sciences ☐ Ecological, evolutionary & environmental sciences

For a reference copy of the document with all sections, see nature.com/documents/nr-reporting-summary-flat.pdf

Life sciences study design

All studies must disclose on these points even when the disclosure is negative.

Sample size	For all cell line xperiments, at least 3 independent samples were used for each analysis.
Data exclusions	No data were excluded from analysis .
Replication	All experiments results showed in this study were performed at least 3 times under independent experimental conditions. All attempts at replication have been successful.
Randomization	Invtro experiments were not randomized but were usually performed in a different format with same treatment condition (e.g., 6-well plates vs. 12-well plates).
Blinding	Blinded to group allocation (control vs. therapy-treated cell lines) during data analysis (SA-β-Gal staining assay, pHi and pHL detection).

Reporting for specific materials, systems and methods

We require information from authors about some types of materials, experimental systems and methods used in many studies. Here, indicate whether each material, system or method listed is relevant to your study. If you are not sure if a list item applies to your research, read the appropriate section before selecting a response.

Materials & experimental systems

n/a	Involved in the study
☒	☐ Antibodies
☐	☒ Eukaryotic cell lines
☒	☐ Palaeontology and archaeology
☒	☐ Animals and other organisms
☒	☐ Clinical data
☒	☐ Dual use research of concern
☒	☐ Plants

Methods

n/a	Involved in the study
☒	☐ ChIP-seq
☐	☒ Flow cytometry
☒	☐ MRI-based neuroimaging

Eukaryotic cell lines

Policy information about cell lines and Sex and Gender in Research

Cell line source(s)	MDA-MB-231 (human triple-negative breast cancer cell line) and MCF-7 (human luminal A type breast cancer cell line) cells were obtained from the American Type Culture Collection.
Authentication	MDA-MB-231 and MCF-7 cell lines were ordered from reliable sources with authentication certification (ATCC,Manassas, VA, US)
Mycoplasma contamination	Cell lines in culture were routinely tested and confirmed to be negative for Mycoplasma contamination.
Commonly misidentified lines (See ICLAC register)	No commonly misidentified cell lines were used in this study.

Flow Cytometry

Plots

Confirm that:

☒ The axis labels state the marker and fluorochrome used (e.g. CD4-FITC).

☒ The axis scales are clearly visible. Include numbers along axes only for bottom left plot of group (a 'group' is an analysis of identical markers).

☒ All plots are contour plots with outliers or pseudocolor plots.

☒ A numerical value for number of cells or percentage (with statistics) is provided.

Methodology

Sample preparation	A total of 3 × 10^5 cells were seeded into each well of 6-well plates and allowed to attach overnight. The cells were harvested after treatment with the therapeutic drugs, followed by washing in phosphate-buffered saline (PBS) and fixation using 70% (v/v) ice-cold ethanol for 1 h (or at 4°C overnight). Cella were washed in PBS, the cells were then incubated with propidium iodide staining solution for 15 min at room temperature before analysis.
Instrument	After staining, cells were analysed on BD LSRFortessa Cell Analyzer (BD Biosciences).
Software	Data were collected using BD FACSDiva Software v8.0.1 (BD Biosciences) and further analyzed by ModFit LT software v5.0 (Verify Software House and GraphPad Prism software v9.4.1 (GraphPad Software).
Cell population abundance	The proportion of live cells was more than 90% of the total number of resuspended cells.
Gating strategy	Cells were selected from cellular debris based on the FSC area/SSC area, singlets then were selected based on the PI-FL2 area/ PI-FL2 Height. Set cells with unincubated PI as a negative control, and adjust the appropriate gate voltage according to the PI fluorescence intensity.

☒ Tick this box to confirm that a figure exemplifying the gating strategy is provided in the Supplementary Information.
